# Defining the Impact of Genetics on Equine Performance and Development of Orthopaedic Disease

**DOI:** 10.3390/ani16121875

**Published:** 2026-06-17

**Authors:** Hannah Chernavsky, Lynn M. Pezzanite, Steve Simske, Chris E. Kawcak

**Affiliations:** 1 Orthopaedic Research Center, Translational Medicine Institute, Department of Clinical Sciences, Colorado State University, Fort Collins, CO 80523, USA; hannah.chernavsky@colostate.edu (H.C.); lynn.pezzanite@colostate.edu (L.M.P.); 2 Department of Clinical Sciences, College of Veterinary Medicine and Biomedical Sciences, Colorado State University, Fort Collins, CO 80523, USA; 3 Immunotherapy Research Laboratory, Translational Medicine Institute, Department of Clinical Sciences, Colorado State University, Fort Collins, CO 80523, USA; 4 Walter Scott, Jr. College of Engineering, Colorado State University, Fort Collins, CO 80523, USA; steve.simske@colostate.edu

**Keywords:** equine, genetics, orthopaedic disease, performance

## Abstract

In equine veterinary medicine, there is a lack of correlation between the structural severity of orthopaedic disease and the perceived severity of presenting signs, resulting in a variable influence on performance, pain and disability. Across equine industries, there is selective pressure through breeding for specific traits, such as the “speed” gene (MSTN) in racing thoroughbreds and the “gait keeper” gene (DMTR3) in pacing breeds, potentially resulting in genetic predispositions that lead to an increased risk of musculoskeletal disorders. With advancements in molecular sequencing techniques, researchers have been able to identify various genes (e.g., ECA, RUNX, and ADAME1) as well as biomarkers influencing performance and the development of orthopaedic disease. In combination, these techniques could be applied clinically in the future to improve welfare and disease management through advanced characterization and treatments to delay the onset of orthopaedic disease.

## Simple Summary

Equine veterinary medicine clinicians are presented with the added challenge, compared to human physicians, that their patients are unable to convey what they are feeling. This leaves determining whether their behavior is related to disease, open to interpretation. The inability of horses to consistently communicate pain and dysfunction requires veterinary clinicians to seek alternate diagnostic strategies to understand and characterize disease conditions. Advanced knowledge of the genetic basis of orthopaedic disease, for instance, may mitigate disease incidence and lead to management strategies minimizing limitations on athletic performance and welfare. This review aims to summarize what is known to date on how genetics can impact the general development of the equine athlete and orthopaedic disease, with the goal of utilizing genetic information to better diagnose and treat patients.

## 1. Introduction

Orthopaedic disease is one of the primary causes of pain in equine athletes, leading to lameness, missed training days, medical management, early retirement, and sometimes extensive and costly surgical interventions [1,2]. Conditions such as osteochondrosis (OC), osteoarthritis (OA) and tendon and ligament injuries can have substantial welfare and economic impacts across all disciplines. Currently, diagnosis relies on a combination of subjective clinical examination and radiographic imaging [3,4,5]. Additional methods such as intra-articular analgesia as well as magnetic resonance imaging (MRI) and computed tomography (CT) can be used to provide clinicians with additional information [1,5,6,7,8]. Recently, behavioural assessment tools, such as the Composite Orthopaedic Pain Scale, which has been found to be an accurate and reliable pain scale for horses experiencing orthopaedic pain, have been introduced as semi-objective assessments [9]. Despite these advances, several limitations remain. Clinical signs as well as imaging do not always reflect structural disease severity, and horses cannot communicate pain or dysfunction, presenting challenges in accurately characterizing disease state and progression.

**Figure 1:**
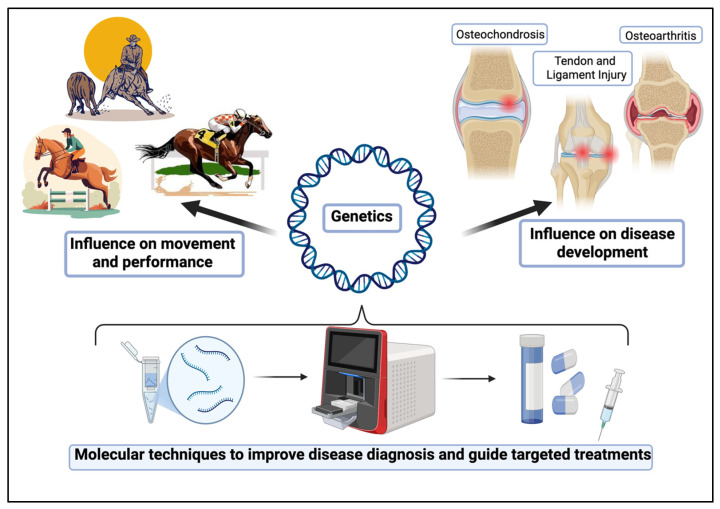
Review of the impact of genetics on equine performance and orthopaedic disease. Figure created in BioRender 2026; Chernavsky. H (Vector images sourced: Vecteezy, Vector Clip Art Illustration, Dreamstime).

Advances in molecular biology and next-generation sequencing techniques have provided new opportunities to investigate the biological mechanisms of both performance and orthopaedic disease [10]. Research involving next-generation sequencing techniques has been able to identify genes that could be related to movement, various orthopaedic diseases and their severity, as well as variability in each athlete’s perception of pain [11]. However, the current knowledge across breeds, disciplines and orthopaedic conditions remains disconnected. Therefore, as summarized in Figure 1, the objective of this review is to summarize what is known to date about the role of genetics in influencing the movement of equine athletes and the development of orthopaedic disease, emerging molecular approaches for disease characterization, and opportunities for integration into clinical practice [11].

## 2. Genetic Basis of Equine Performance and Orthopaedic Disease

***Impact of Performance-Based Selective Pressure on Genetic Diversity***—Today, technological advances enable breeders and owners to refine the type of athlete they are aiming to produce. However, these innovations potentially increase vulnerability to negative effects on genetic diversity and predisposition to the development of orthopaedic disease [12]. The effects of selective breeding can be appreciated in industries such as the thoroughbred racing industry and the American Quarter Horse Association (AQHA).

To expand on this point, the lineages of racing thoroughbreds demonstrate strong selective pressure. In North America, the thoroughbred industry is governed by the Jockey Club. For a foal to be eligible for registration, it must be a product of live cover, a rule that was introduced to prevent the dominance of a single sire [13]. However, recent studies evaluating the influence of sires over various populations of racing thoroughbreds have revealed that despite efforts to increase genetic diversity, it is still limited. As an example, one study identified that in a population of 10,118 registered thoroughbreds worldwide, four of 305 prominent sires were found to have significant influence over this entire population, three of which trace back to North America [14]. These findings indicate that while there are multiple branches of racing across continents, there is still limited dilution within the gene pool of registered thoroughbreds. Evidence of this narrowing can also be seen at the genomic level. Whole-genome sequencing was performed on a separate group of North American Thoroughbreds (n = 185) where horses were split into two groups based on birth year (Group 1: 1965–1985, Group 2: 2000–2020). Analyses revealed that horses born in Group 1 demonstrated increased genetic diversity in comparison to those born in Group 2 [15]. This decline could be explained by a consistent decrease in registered sires, dams and foals over the last 30 years. Looking at foal crop alone, ~40,000 foals were registered in the United States in 1990 compared to 16,000 in 2025. Similar trends have been noted with the number of registered stallions in 2010 being 2791, which dropped to 804 in 2025 [13]. Declining genetic diversity may increase the risk of concentrating deleterious alleles within a population, potentially increasing susceptibility to inherited disorders including orthopaedic conditions.

While the objective of breeding within the thoroughbred industry gravitates towards producing the fastest horse, the AQHA is unique in that there are multiple disciplines and genetic traits that are targeted. Unlike the Jockey Club, the AQHA allows for multiple methods of breeding. This regulation has produced both an increase and a decrease in genetic diversity [16]. Until recently, the AQHA had a regulation stating that when a registered sire died, any banked semen could be used for only two years following their death. However, this applied only to stallions born after 2015. Semen stored from stallions prior to 2015 could be used indefinitely [16]. This regulation alone could have several implications for the gene pool. One, from the domination of older sires due to their continued use, leading to their prominence within a population without the same ability for younger stallions to contribute. Additionally, this regulation could result in a rapid increase in the use of deceased successful sires’ semen to produce as many foals as possible. This could have a few negative implications. For example, if a sire were to die at a young age prior to knowledge of or development of an orthopaedic condition, the foal crop from this sire now has an unknown risk of orthopaedic disease development. In 2026, this regulation was overturned, allowing extended use of frozen semen and embryos. Unlike thoroughbreds, Quarter Horses participate in multiple events ranging from racing, where speed is the main goal, to cutting where agility is a major factor in their success. The Quarter Horse breed has been found to have what is considered substantial genetic diversity relative to some other breeds; however, when investigating each discipline, diversity experiences a significant reduction [17,18]. However, when diving deeper into single disciplines within the breed, effects similar to the thoroughbred racing industry can be seen, as breeders target the use of successful sires in each discipline, thus concentrating the gene pool.

Although industries such as the Jockey Club and AQHA have placed stipulations on breeding practices to protect the genetic diversity of the breed, there is evidence of genetic narrowing across both industries. Selective breeding used to enhance specific traits has led to the repeated use of successful bloodlines, with concurrent impacts on genetic diversity, concentrating the frequency of specific genetic variants, sometimes deleterious, and exposing breeds to the development of orthopaedic disease. Consequently, identifying the genes responsible for desirable performance or athletic traits has become a major focus of equine genetic research. These investigations have led to the identification of genes influencing athletic traits such as muscle development and locomotion, providing further insight into the biological impacts of selective breeding.

***Genetic Influence on Movement Types and Performance***—Advancements in breeding technology, in combination with continued advancement of the field of genetics, have allowed breeders access to improved specificity in the selection of traits such as speed, jump height, overall movement and even behaviour. A survey was distributed to members of the World Breeding Federation of Sport Horses, aimed at determining the most important objectives of breeding in this industry [19]. Movement, conformation and gaits, alongside performance in dressage and show jumping were found to be the most valued and common objectives. While not the main objectives, some breeders included soundness, behaviour and fertility [19]. These breeding objectives have driven efforts toward the identification of genetic variants associated with desirable athletic phenotypes, as summarized in Table 1, leading to the discovery of genes linked to traits such as gait and muscle development.

Broadly, various performance and gait-related traits have been found to be influenced by genetic variations, leading to changes such as musculoskeletal development and neuromuscular control. Two genes that have become well-characterized and associated with equine performance are myostatin (MSTN) in racing thoroughbreds and Quarter Horses, and DMTR3 in standardbreds [20]. In the thoroughbred racehorse, polymorphisms in the MSTN gene, also referred to as the “speed gene”, have been directly linked to this breed’s race distance aptitude. MSTN is a gene that encodes for myostatin, a paracrine hormone that is skeletal muscle-specific and plays an important role in muscle development. Normal expression of MSTN inhibits muscle proliferation, creating a stopping point for muscle development. Mutations to MSTN can have several effects, such as decreasing muscle proliferation or, conversely, mutations leading to an absence of inhibition on myostatin, resulting in extreme skeletal muscle hyperplasia, a condition known as ‘double muscling’ [21,22,23]. In the racing thoroughbred, there are two common MSTN variants. The first is a single-nucleotide polymorphism (SNP) that is related to race distance aptitude, where horses with C/C paired alleles are better suited to short-distance races, C/T paired alleles for mid-distance, and T/T paired alleles for long-distance races. The T allele is considered the ancestral gene, with the C allele reported to have been introduced to the breed by a single mare 300 years ago [20,22,23,24,25,26]. The second MSTN variant can be found as a SINE mutation on the gene’s promotor. In horses that are C/C allele-paired, the SINE insertion can be found, while it is absent in horses with T/T allele pairs. This SINE insertion has been reported to be associated with reduced myostatin expression between the MSTN genotype and race distance, further contributing to the race distance aptitude of the allele variants [22,23,24,26]. The association between the MSTN genotype and aptitude towards race distance demonstrates how genetic variation can directly influence physiologic changes, such as muscle development, and provides a foundation for how specific genetic markers can be linked to measurable performance outcomes.

While MSTN primarily influences performance through the regulation of muscle development, other genes affect performance and athleticism through changes in locomotor control. Nicknamed the “gait-keeping” gene, the doublesex and mab-3-related transcription factor 3 gene (DMTR3) has been identified as affecting locomotor patterns. In mice, a gene knockout was performed, confirming a significant effect on locomotion and limb coordination [27]. This gene encodes a vital transcription factor that is involved in limb coordination. The occurrence of a premature stop codon was found to be associated with a significant change in gait performance [27,28]. The normal variant of this gene will produce the gait known as a “trot” where the hind and fore limbs move together in diagonal pairs. However, the occurrence of a premature stop produces the gait called a “pace” where the hind and forelimbs move in lateral pairs [27,28,29,30]. Today, some populations such as the American Standardbred, Tennessee Walking Horses and Missouri Foxtrotters have fixed polymorphisms leading horses to primarily pace, which is desirable in certain forms of racing. Populations such as Norwegian and Swedish Standardbreds are nearly fixed, while the rest of the populations still retain polymorphisms [20,27,29,30]. This demonstrates how selective pressure for pacing has led to the elimination of select genotypes to produce a specific type of athlete. Together, MSTN and DMTR3 demonstrate how performance traits can arise from various biological pathways, including musculoskeletal development and locomotor regulation.

When examining discipline-oriented selective pressures, as in selection for “pacing”, the influence of performance-associated genes becomes more evident. As stated previously, the Quarter Horse population demonstrates increased genetic diversity in comparison with other breeds where high selective pressure is experienced (e.g., racing thoroughbreds). However, when broken into individual populations by discipline, there is a reduction in diversity. A genome-wide association study mentions both myostatin (MSTN) and DMTR3 as genes of significant contribution towards the performance of Quarter Horses [31]. However, unlike standardbreds where the presence of changes on an allele on the DMTR3 gene is desired, this presence in combination with subjective assessment suggests a negative effect on the overall performance of the Quarter Horse [31]. CKM has also been introduced as a gene that may contribute to changes in the musculoskeletal and nervous system, leading to downstream effects on the horses’ movement, enhancing their performance in their events [31,32]. These findings further expand on how selective breeding can shape the frequency of performance-associated alleles within a population according to the athletic demands of a particular discipline.

Although several genes have been reported to be associated with movement and athletic performance, the expression of these traits is not always influenced by genetics alone. Rider influence, as well as environmental factors such as peak performance age, may also influence outcomes. In comparison to thoroughbreds and Quarter Horses, warmbloods do not reach their potential in high-level events until they are older, increasing the generational gap and leaving room for other proven athletes to enter the gene pool [33]. While this may be beneficial for the genetic diversity of the breed, it is viewed as economically inefficient. In addition to genetic influence, movement characteristics can be modified by training methods or rider-mediated biomechanical changes and can be defined as “rider influence” [33]. For example, in industries such as reining, horses are trained and ridden with a low head carriage compared with those competing in dressage, which in turn can influence the overall movement of the horse. Due to these variations, industries such as the warmblood industry have introduced the idea of evaluating horses at a younger age alongside their dams, such as in foal shows and under free movement to eliminate rider influence, to determine their phenotypic heritability [33,34]. Comparisons of free versus ridden movement demonstrated increased heritability in free movement, indicating that while breeders may select for heritable traits, a rider in this industry can strongly influence their athlete’s general movement and performance [33,34]. This introduces the idea that performance traits are a complex combination of genetic, physiologic and environmental factors [20]. Collectively, these findings demonstrate how genetic variation can produce measurable changes in locomotion and performance. With the ability to identify genetic markers associated with specific phenotypes, investigation has been prompted to determine if similar approaches can be pursued to identify genes influencing susceptibility and development of orthopaedic disease and variability in progression.

***Durability Index in the Thoroughbred Horse***—While the development of performance traits such as speed, gait and overall athletic ability are targets of selective breeding, longevity and soundness are important considerations as well, specifically in industries where there is an elevated occurrence of musculoskeletal injury. In some industries, such as the thoroughbred industry where there is increased public exposure, indices for performance traits such as durability and offspring success and longevity have been created [35]. Artificial selection to create the best racehorse has led to specific signatures in the thoroughbred genome that are exercise-adapted, favouring traits leading to enhanced performance and speed, which can be evaluated through heritability estimates. Briefly, heritability estimates are a quantitative measure of assessing genetic variation in livestock. This measurement accounts for genetic traits, while all other factors are attributed to the environment [36]. Previous studies have demonstrated race performance to be a heritable trait; however, shortcomings arise due to no objective measure of heritability [36,37]. However, the Grayson Jockey Club has introduced a durability index to rank the top 100 stallions by progeny earnings, by the number of progeny starts and percentage of foals that raced. Since 1960, the average number of starts each horse enters has decreased from 11 to less than seven. The goal of this index is to highlight sires with successful progeny in multiple race starts, to help introduce genetic lines with a proven history into the breed [38]. By emphasizing career longevity and race frequency in addition to earnings alone, the durability index reflects increasing interest in identifying heritable factors associated with soundness and athletic performance.

Additionally, whole-genome sequencing (WGS) has assisted in identifying specific genes that may lead to improved performance or hardiness in the thoroughbred horse [39]. For positive selection, genes related to reproduction and exercise-related physiology experience the strongest selection pressure. For example, overrepresentation of focal adhesion complex genes suggests these genes have played a significant role in shaping the modern thoroughbred [39]. Through genome-wide association studies (GWAS), MSTN has also been highlighted as a key gene in performance [36]. Selective breeding initiatives focused on optimizing athleticism and durability may inadvertently introduce and perpetuate genetically-linked orthopaedic conditions as well. As a result, it is important to understand the genetic foundation of both orthopaedic disease to balance athletic success and long-term soundness. Combined, the investigation of performance as well as athletic durability and longevity underpin the capability of genetic analyses to identify biologically meaningful traits. To build on this, similar approaches have been applied to the development of orthopaedic disease, with the aim of understanding disease susceptibility, progression and potential diagnostic application.

***Genetic Influence on Orthopaedic Disease Development***—Molecular technology advances have supported the concept that genetics play a role in the performance of equine athletes. However, the influence of genetics reaches beyond athletic performance and can also contribute to the predisposition or susceptibility to orthopaedic disease. Similar to athletic and performance-associated traits, several genetic variants have been associated with orthopaedic conditions such as osteochondrosis (OC), osteoarthritis (OA) and tendon and ligament injuries, as summarized in Table 2. Identification of these disease-associated genes has aided in improving understanding of disease development and provided a foundation for the future development of genetic and molecular diagnostic tools.

Osteochondrosis is believed to result from abnormal endochondral ossification, specifically caused by abnormal or mutated chondrocytes. This disease commonly affects joints such as the fetlock, hocks and stifles [40,41]. Currently, no single cause of OC has been identified; however, genetic predisposition and risk have been investigated. Across various breeds of horses, rates of prevalence and heritability differ [12]. Radiographic surveys of the fetlock in thoroughbreds and warmbloods demonstrate a 7.2 to 14.9% prevalence rate, respectively, while French Trotters showed a 32% prevalence rate. Coldbloods, represented by heavy draft breeds such as Percherons and Shires, presented with the highest rate of 53.9%. Similar patterns can be found when evaluating stifle and hock OC prevalence [42,43,44,45,46,47,48,49]. Heritability also demonstrated a similar trend with lower rates in thoroughbreds (0.00–0.16) compared to trotters (0.11–0.45) [42,43,44,45,46,48,49]. These patterns in development can be attributed to multiple factors including the workload of each horse, discipline, or weight and bone structure (e.g., thoroughbreds versus draft breeds). Additional studies have sought to identify specific genetic variants contributing to OC development. Methods used included searching for single-nucleotide polymorphisms (SNP), microsatellites, or repetitive DNA sequences to seek out OC associated risk loci [48]. Various quantitative trait loci have been identified on *Equus Caballus* autosome (ECA), which has been further supported by the identification of several OC-associated SNPs and single-nucleotide variants (SNV) on ECA [46,50,51,52,53,54,55,56,57,58,59]. Additional genes such as insulin-like growth factor-1 (IFG1), matrix metalloproteinase-13 and 3 (MMP13, MMP3), runt-related transcription factor 2 (RUNX2) and parathyroid hormone-related protein (PTH-RP) have also been linked to roles in the development, maturation and ossification of cartilage and bone [60,61,62,63,64]. Several of these highlighted genes play a role in cartilage maturation and skeletal development, providing a biological foundation for their association with OC development. It is important to note that additional environmental factors such as growth rate, weight and exercise are also contributors to disease development. In combination, downstream effects of the presence or alterations of these identified genes, as well as environmental contributors, could lead to the occurrence of developmental diseases such as OC.

While both OC and OA can affect joint health and athletic performance, they represent distinct biological processes. Unlike OC, OA is more commonly seen in later stages of life. Considered a leading cause of lameness, lost training days and reduced athletic ability, affecting 60% of horses aged over 15, OA is characterized by the degradation of cartilage, synovitis, osteophyte formation and subchondral bone sclerosis [2,65,66,67,68,69,70,71,72]. While the direct cause of OA is unknown, its development can be attributed to trauma and repetitive stress injury. On a molecular level, increased production of matrix metalloproteases as well as pro-inflammatory cytokines, nitric oxide and prostaglandins can be found [71]. When using PCR to evaluate differential expression of genes in peripheral white blood cells of horses affected by OA, ADAMEC1, GRP, HCST, HUNC-93a and RRM2 were found to be significantly differently expressed in horses with induced OA [73]. RNA-Seq and PCR assays were used to study a greater number of genes, revealing 42 genes linked to the extracellular matrix, as well as degradative proteases, growth factors, and cytokines in cartilage sampled from older versus young horses. RUNX2, COL2A1, COL1A2 and IL1β have also demonstrated increased differential expression in tissues affected by OA [74]. While these identified genes can increase susceptibility, influence inflammatory responses, cartilage metabolism or tissue repair, it is important to note the contribution of the equine athletes’ environment as well. Mechanical loading, history of injury and general management contribute to disease development in tandem with genetic susceptibility. Although variations in differential gene expression do not explicitly indicate predisposition to the development of OA, their consistent association with disease-related progression suggests their potential utility as molecular biomarkers for disease detection, monitoring and treatment.

The influence of genetics on orthopaedic disease does not stop at articular cartilage or bone development, but also extends to tendons and ligaments, which play a crucial role in locomotion and the development of orthopaedic disease. While all breeds are susceptible to injury and genetic predisposition to disease development, racing thoroughbreds carry a high risk of tendon and ligament injuries, specifically those to the superficial digital flexor tendon and suspensory ligament [75,76,77]. When measuring heritability of risk of suspensory ligament and tendon injuries alongside fracture and OA risk, a relatively low risk was found (0.01–0.20). However, further investigation indicated a positive genetic correlation with the development of OA and suspensory ligament injury, suggesting suspensory ligament injury as a risk factor for OA, or vice versa. It was also noted that when comparing thoroughbreds originating from Europe versus Australia or other continents, European horses had a reduced risk, and horses originating from North America carried an even lower risk [75]. While insight on heritability can be informative to breeders, a population of National Hunt thoroughbred racehorses was used to identify specific genes associated with risk of superficial digital flexor (SDF) tendinopathy. Horses found to be heterozygous for TNC polymorphism carried a decreased risk of SDF tendinopathy versus those who were homozygous. Additionally, horses that are homozygous for the COL5A1-01 allele carry a three times higher risk of developing SDF tendinopathy compared to those with the homozygous wild-type allele [78]. These assessments demonstrate the ability to use genetics to identify potential disease development across musculoskeletal tissues, supporting their potential use as molecular biomarkers to monitor athletic well-being and disease development.

As noted, several genes and genetic variants have been identified as associated with the development of orthopaedic disease. Knowledge of these disease-associated genes can provide additional information to breeders when making decisions in producing athletes, or owners when pursuing a course of action for an injured athlete. However, it is important to note that these associations with orthopaedic disease do not imply disease inevitability. Orthopaedic diseases are complex conditions which are influenced by a combination of genetic and environmental factors. And due to the complexity of disease development, owners may not always notice early stages of development of orthopaedic disease in their athletes due to variability in outward presentation of pain. These factors provide a foundation to utilize the identification of disease-associated genes to explore beyond traditional assessment methods. Continued advancement in molecular technologies provides the ability to utilize candidate genes as a molecular diagnostic approach with the capabilities of identifying disease susceptibility, improving disease characterization and assisting in the evaluation of disease severity and progression.

## 3. Structural Disease Severity and Progression Differs Between Patients

The development, progression, and clinical presentation of orthopaedic disease are variable between patients. While several genes have been associated with susceptibility to orthopaedic disease, its progression and clinical presentation are highly variable among individual equine patients. This variability makes it difficult to characterize the disease state when relying solely on traditional diagnostic methods (e.g., clinical assessment and diagnostic imaging). As a result, molecular biomarkers have become increasingly utilized to predict the likelihood of disease or to characterize its progression. In tandem, genetic and molecular biomarkers can aid in identifying a genetic predisposition to the development of orthopaedic disease or indicate disease severity, including cartilage turnover and bone remodelling at the time of sampling. Molecules such as CPII, C2C, Il1β and TNFα have been highlighted in recent studies as biomarkers indicative of disease state, as they reflect the current state of the affected tissue and the release of inflammatory cytokines responsible for the degradation of bone and cartilage. Currently, biomarker applications have been found in areas such as early detection of orthopaedic diseases such as OC, monitoring degenerative diseases like OA, and predicting soft tissue injuries.

While genetic studies may aid in the identification of horses with increased susceptibility to OC, biomarkers can add an additional layer of information regarding current disease development and progression. A study performed for early-stage detection of osteochondrosis utilized a biomarker panel representative of skeletal metabolism. These biomarkers are believed to reflect changes in bone and cartilage turnover, age, growth, feeding level and the occurrence of OC. Osteocalcin concentrations, alongside c-propeptide of type II procollagen (CPII)/C2C ratios at two and 20 weeks respectively, demonstrated a strong positive correlation with OC diagnosed radiographically at five and a half months. Similarly, a strong correlation with osteocalcin and radiographs detecting OC at 11 months was found; however, no changes between CPII and C2C ratios were noted [79]. The findings suggest that biomarkers have the potential to serve as early indicators for horses at risk of developing OC prior to clinical signs. Tools such as this could allow intervention at earlier timepoints, such as during skeletal growth, where modifications to general management may be most effective.

Biomarkers have also been utilized in the diagnosis of OA; however, several studies have used an acute experimentally induced model and a short timeline that is not always representative of true disease development [6,79,80,81,82,83,84,85,86,87,88,89,90,91,92,93]. A longitudinal study on standardbred racehorses was performed with the purpose of examining how pro-inflammatory cytokines and structural biomarkers might change over time, and whether they can serve as an effective tool to categorize disease progression of post-traumatic OA. When observing temporal changes in cytokines and structural biomarkers over the course of training, CTXII was significantly elevated in year two, and COMP was significantly elevated in year three. Similar trends were seen in inflammatory cytokines such as Il1β in year two, and IL6 and TNFα in year three. This study presents preliminary evidence for the ability to stage the development of OA; however, these results are limited by the fact that some horses in this study were receiving disease-modifying treatments [94]. As with OC, biomarkers have the potential to work in tandem with common diagnostic methods and provide an objective measure of cartilage or bone degradation. More detailed information for clinicians can improve assessment as well as treatment methods, particularly in early stages of the disease where radiographic changes are not yet visible.

The risk of developing tendon and ligament injuries has also been assessed through the use of biomarkers. Studies performed using thoroughbred racing horses determined that biomarkers such as glycosaminoglycan and CPII can act as predictors of injury [95]. Patterns in similar biomarkers in an alternate study also demonstrated an ability to be used as a tool for screening horses prior to the occurrence of injury [96]. However, as with OC and OA, tendon and ligament injuries can present with different grades of severity. Unfortunately, evidence for the use of biomarkers when grading tendon or ligament lesion severity is limited [97]. However, the potential downstream ability to identify an athlete with an increased risk of injury prior to clinical manifestation represents an important step towards preventative disease management and highlights the value of molecular markers as a method of preventative medicine.

Combined, these studies demonstrate the potential use of biomarkers to objectively measure pathological processes such as cartilage turnover, inflammation and bone remodelling when traditional diagnostic methods cannot. Although many of the proposed biomarkers are limited to experimental settings and require further validation, they provide a pathway to developing diagnostic methods involving molecular markers for improved disease characterization. Used alongside genetic information, biomarkers and transcriptomic analysis have the potential to contribute towards the development of diagnostic panels capable of identifying at-risk horses, disease state and monitoring disease progression. Translational use of this approach is variable; however, further potential refinement of a bedside diagnostic panel to assess disease progression may improve treatment techniques in both veterinary and translational medicine.

## 4. Future Directions

Although there have been significant developments and advances in studies of equine genetics, there is limited translation of these discoveries into clinically applicable diagnostic tools. Currently, diagnostic methods for equine orthopaedic disease rely heavily on the use of subjective examinations, diagnostic imaging and, more recently, semi-objective tools such as ethograms. However, they remain limited in their ability to accurately identify and scale disease severity and pain. Advances in genetic analyses have demonstrated that both performance traits and the development of orthopaedic disease are influenced by heritable factors. However, selective breeding practices to create top-tier athletes have introduced a trade-off of reduced genetic diversity and potential increased disease risk. While various genetic associations and biomarker studies have successfully identified their involvement in orthopaedic conditions such as OC, OA and tendon and ligament injuries, there has been limited validation of their clinical application. Emerging evidence also suggests variability in pain sensitivity and expression may be genetically linked as well, further complicating orthopaedic disease in equine athletes. This complexity is also supported by variability in disease progression between athletes. Collectively, these challenges highlight an opportunity to develop molecular diagnostic approaches capable of improving the diagnosis and characterization of orthopaedic disease and clinical decision-making.

Given the information discovered thus far, genetics play an increasing role in the diagnosis, grading and treatment of several diseases, and are an increasing topic of interest within the last few decades [98]. While biomarkers are currently explored as methods of diagnosis and grading, they do not always accurately represent the disease state that can be visualized within the joint. A recent study aimed to determine whether subjective lameness exams or disease-associated biomarkers could predict disease severity (OA); both did not demonstrate any ability to predict disease severity. However, this study was limited to the use of only two biomarkers. An additional analysis was performed in this study utilizing RNAseq and transcriptomics as a measurement for predicting disease severity. This analysis provides a window of opportunity to further explore the use of genetics and next-generation sequencing as a method of early diagnosis, measuring disease progression, as well as guiding treatment.

A similar gap exists in human medicine where the way a patient presents subjectively and their actual disease state vary [99]. While subjective questionnaires and gait examinations as well as radiographs and more advanced diagnostic imaging are used as diagnostic methods in humans, veterinary medicine is further inhibited by a patient’s inability to directly communicate what they are experiencing. Although arthroscopy is available as another diagnostic method in both human and veterinary medicine, it is far more invasive [99]. As an advancement, human medicine is turning to the use of genetic and biomarker panels to better assess disease states [98,100,101,102,103]. While additional genetic research is needed, synovial fluid biomarkers have shown promising results in areas such as diagnosing knee OA. Due to its easy access, synovial fluid has become increasingly popular as a sample to collect for biomarker analyses. A meta-analysis aimed at determining the efficacy of SF biomarkers proposed for analysis of knee OA using the BIPEDS classification identified multiple biomarkers such as IL-6, IL-8, C4S, Leptin, TIMP-1, MMP-1/3, VEGF and TNF- α. Additionally, from the biomarkers assessed, “diagnostic” and “burden of disease” categories from the BIPEDs classification were most represented [103]. This study provides preliminary evidence for the use of biomarkers to diagnose OA, which can be further enhanced through the integration of disease-associated genetic markers. To build upon biomarker-based approaches, advances in transcriptomics and proteomics, for instance, may allow investigation of broader molecular signatures that may be associated with disease susceptibility, progression and severity.

Next-generation sequencing, such as transcriptomics and proteomics, has also provided insight into the development of orthopaedic diseases such as OA [104,105]. Utilizing a biobank in the UK, a large-scale proteomic analysis of plasma samples from OA patients was performed. Analyses were able to identify plasma proteins such as COL9A and CRTAC1 as significant predictors of OA, with deviations beginning up to ten years prior to onset [105]. Recently, a translational genomics study of OA was published, assessing multiple genome-wide association studies resulting in almost two million patients reported. This study found multiple genes to be involved in the development of OA, such as ALDH1A2, ADAMTSL3, COL27A and IL11 [104]. As similar studies emerge, this information can be refined and continued to be applied to the development of diagnostic panels utilizing transcriptomics and biomarkers. Ideally, these tests will also be developed in veterinary medicine, helping to bridge the gap between patient presentation and diagnosing disease severity. Overall, advances in next-generation sequencing and discoveries in multi-omic studies have identified candidate genes and proteins capable of assessing OA onset prior to clinical presentation, supporting the development of diagnostic panels integrating the use of transcriptomics or proteomics alongside biomarkers to improve early detection in both human and veterinary medicine.

Further development of molecular diagnostic panels integrating genetic, transcriptomic, and systemic biomarkers represents a potentially clinically applicable opportunity moving forward. Transcriptomic analyses have suggested the ability of differential gene expression to provide additional information regarding disease severity that traditional diagnostic methods alone cannot identify. Similarly, human medicine is turning to the use of genetic biomarker panels to better assess disease states and identify molecular signatures years prior to disease onset. As these technologies continue to evolve, the development of equine-specific molecular panels can allow for earlier disease detection, improvement in grading disease severity, and monitoring treatment response in horses affected by orthopaedic disease. While these tests do not yet exist, future efforts should prioritize the validation of candidate genes as well as the identification of disease-specific molecular signatures and the development of clinically applicable diagnostic panels. Through the integration of genetic and molecular information with traditional clinical assessments, veterinary medicine can move towards more objective approaches for identifying disease susceptibility, characterizing disease severity, monitoring treatment response and improving welfare in horses affected by orthopaedic disease.

## 5. Conclusions

As technology advances, the field’s appreciation of the role that genetics play in the movement and development of orthopaedic disease in the equine athlete has increased. Multiple genes have been identified to influence performance and risk for disease onset. In the future, advancement of the application of molecular techniques in equine practice with the development of improved genetic or biomarker panels could better inform owners and clinicians, leading to a reduction of disease incidence, earlier diagnoses and individualized treatment.

## Figures and Tables

**Table 1:** Summary of studies reviewed introducing genetic effects on movement.

Investigator	Reference	Breed	Paper Type	Study Type	Sample Source	Main Findings
Koenen et al. (2014)	[19]	Warmblood	Original Research	Survey	44 Breeding organizations—Members of WBFSH	Main breeding objectives focused on gait, conformation and performance in dressage and show jumping. Secondary objectives focused on overall health, soundness, behavior and fertility.
Sharma et al. (2025)	[20]	Multi-breed	Review	N/A	N/A	Genes such as DRD4 and COMT were associated with behavior traits such as trainability. MSTN and KEAP1 were associated with muscle function and oxygen metabolism. Athletic traits developed through complex genetic interactions as well as genetic variants due to environments adaptations.
Aiello et al. (2018)	[21]	Multi-species	Review	N/A	N/A	Myostatin signaling pathways maintain control over skeletal muscle development. Mutations in the MSTN gene can lead to double muscling in popular livestock animals such as cattle and sheep. MSTN polymorphisms in horses have been identified to be associated with racing phenotypes as well as muscle fiber proportions.
O’Hara et al. (2021)	[22]	Thoroughbred	Original Research	ELISA myostatin concentration analysis PCR genotyping for SINE insertion of MSTN promotor	Blood and serum samples from 179 racing thoroughbreds from a single facility.	Homozygous SINE mutation horses presented with lower serum myostatin concentrations than heterozygous horses. Heterozygous SINE mutation horses presented with an decreased concentration of serum myostatin than normal horses. Determined as association between the presence of a SINE mutation in MSTN and optimal race aptitude.
McGivney et al. (2012)	[23]	Thoroughbred	Original Research	qRT-PCR to target myostatin gene	Muscle biopsies from the Gluteus Medius from 60 untrained thoroughbred yearlings and 33 samples from trained thoroughbred yearlings	MSTN is significantly associated with the phenotypes of thoroughbred racing horses. MSTN genotypes can influence the downstream gene expression of MSTN. C/C cohort was found to have the highest levels of MSTN mRNA, while the T/T cohort had lower levels of mRNA, reflecting their short- and long-distance racing aptitudes.
Rooney et al. (2018)	[24]	Thoroughbred	Original Research	PCR-based targeted sequencing (MSTN)	Skeletal muscle biopsies of thoroughbred horses in one training yard under management of single trainer	SINE insertion MSTN polymorphism adversely affected transcription initiation. Downstream effects of myostatin protein production occurred. SINE insertion provided foundation for variation in race distance aptitude in thoroughbred horses.
Bower et al. (2012)	[25]	Multi-breed	Original Research	MSTN SNP genotyping (PCR)	Modern horse (whole blood or hair, n = 593) Modern elite thoroughbred (n = 330, whole blood or hair) Historical Thoroughbred Stallions (bone sample, n = 12) Other equids (n = 42, whole blood or hair)	High frequency of C-variant on the myostatin gene in modern-day thoroughbreds, contributing to genotypes best suited for short-distance racing. C-allele arose from a single introduction during foundation stages of thoroughbreds. C-allele was determined to be rare among top horses in the 18th and 19th centuries.
Hill et al. (2012)	[26]	Thoroughbred	Original Research	MSTN SNP genotyping (PCR), GPS tracking	Thoroughbred racehorses from a single training yard (n = 85), whole blood	Presence of C allele at the MSTN gene improved race speed when comparing C/C, C/T and T/T genotypes. C/C and C/T genotypes outperformed T/T in speed. C/C genotypes presented with increased mean distance and increased maximum velocity compared to T/T.
Andersson et al. (2012)	[27]	Icelandic, Swedish Trotters, DMTR3 null mice	Original Research	GWAS, DMTR3 SNP genotyping (PCR), RtqPCR (mice)	Icelandic and Swedish trotters in Sweden DMTR3 null mice	Premature stop codon on DMTR3 gene had significant effects on locomotion pattern in horses. DMTR3 mutation resulted in “pace” movement measured samples of Icelandic and Swedish Trotters. Genetic control over locomotor function was confirmed by DMTR3 gene knockout mice.
Regatieri et al. (2016)	[28]	American Saddlebred	Original Research	PCR-RFLP DMTR3 genotype	Hair samples from 242 randomly selected Saddlebred horses Typed from two variants of DMTR3	Presence of a premature stop codon in the DMTR3 gene results in changes in locomotion causing a horse to race. There were no differences in frequencies when observing five gaited horses compared to other American Saddlebreds.
Cothran et al. (1987)	[29]	American Standardbred	Original Research	Genetic marker typing	Group 1: 856 pacers, 371 trotters Group 2: 1227 pacers, 600 trotters	Gait was an outcome of assortive mating in American Standardbreds. Trotters and pacers shared common alleles at 20 of the 23 measured loci; however, there were significant differences in allele frequencies at 21 of the 23 measure loci. Genetic differences between standardbred trotters and pacers were greater than differences between some distinct horse breeds.
Promerova et al. (2014)	[30]	Multi-breed	Original Research	DMTR3 SNP genotyping, PCR and Sanger sequencing	Genetic Laboratory Banks (whole blood or hair samples, n = 4396 representing 41 breeds)	68 of the sampled 141 breeds demonstrated a DMTR3 mutation. Mutation is not breed-specific and can be found worldwide but has been selected for in specific industries. >50% presence of DMTR3 mutation was used to define a breed as “gaited”.
Pereira et al. (2016)	[31]	Quarter Horse	Original Research	MSTN and CKM genotyping (PCR-RFLP, sequencing)	Quarter horse whole blood (n = 364)	Limited variability in MSTN gene indicated large selective pressure towards homozygous C/C variants. Heterozygous DMTR3 gene had a negative effect on racing performance in Quarter Horses.
Avila et al. (2018)	[32]	Quarter Horse	Original Research	SNP70 genotyping	Quarter Horse performance horses (n = 143), hair samples	Positive selection was identified on all 31 autosomes tested. Genes associated with metabolism, skeletal muscle development and central nervous system development were identified in specific regions of interest for breeders and development of athletes.
Ducro et al. (2007)	[33]	Dutch Warmblood	Original Research	Heritability analysis	KWPN Studbook Entries (1992–2002)	General movement traits demonstrated moderate to strong genetic correlation. Heritability of combined traits varied (e.g., free jumping and dressage demonstrated weak to moderate genetic correlation).
Becker et al. (2001)	[34]	German Warmblood	Original Research	Heritability analysis	Oldenburg Horse Breeders Society Mare Performance Test (2000–2008)	Heritable traits demonstrated during free movement or under rider may not be interchangeable. Greater specificity of trait definition for gait evaluation under saddle or free movement could assist in breeding selection.
Bennet et al. (2026)	[35]	Thoroughbred	Original Research	Retrospective Cohort—Equine Injury database (EID) and risk factors	Race starts (n = 3,851,659) of horses born after 31 December 2006 (n = 25,840) on tracks reporting to EID between 2009 and 2023 on tracks in US and Canada (n = 115).	Rate of 1.49 fatal musculoskeletal injuries for every 1000 race starts. 20 of the 97 measured risk factors presented with statistically significant association with increasing or decreasing odds of musculoskeletal injury.
Bailey et al. (2022)	[36]	Thoroughbred	Review		Heritability and GWAS studies	Measures such as race time and money earned demonstrated variability in heritability but were poor indicators of success. Myostatin genomic variants were contributors to race success.
Langlsi and Blouoin (2007)	[37]	Thoroughbred and French Trotters	Original Research	Race outcome aggregation and available genetic information	Society for the Promotion of French Horse Breeding Database	Race earnings and race starts provided limited ability to determine outcomes. Calculation measures such as log transformation better account for measured horses with zero race earnings and improved heritability analyses.
Grayson Jockey Club Research Foundation (2026)	[38]	Thoroughbred	Original Research	Durability and Soundness Index	Top 100 stallions by progeny earnings in 2025	Stallions with progeny entering higher number of races and race earnings are thought to be of better durability and soundness.
Gu et al. (2009)	[39]	Thoroughbred	Original Research	Microsatellite markers	Thoroughbred samples from available repository (n = 815) Non-thoroughbred samples available from repository (n = 52)	Genomic regions under selection pressure were linked to metabolism and muscle function. Key pathways supporting performance included insulin signaling, lipid metabolism and muscle structure. Thoroughbreds served as a strong model for studying exercise adaptation and metabolic disease prevention at the molecular level.

**Table 2:** Summary of studies’ genes contributing to the development of orthopaedic disease.

Investigator	Reference	Disease	Breed	Paper Type	Study Type	Sample Source	Main Findings
Serteyn et al. (2010)	[40]	Osteochondrosis	Belgian Warmblood	Original Research	Digital gene expression analysis and Rt-PCR	Group 1: Control, n = 11 Group 2: OC-affected, n = 11 Whole blood collections	Metabolic pathway analysis revealed dysregulation of various signaling pathways relating to cartilage formation and repair. Significant differences were found between genes associated with carbohydrate diet, inflammation, and abnormal insulin between control and OC-affected horses. Significant differences were found between control and OC-affected horses in DGE analysis of transcript profile of leukocytes.
Distl (2013)	[41]	Osteochondrosis	Multi	Review	Reviewing GWAS, SNP, Whole genome scans (microsatellite)	Studies utilizing GWAS, SNP, NGS, microsatellite	Microsallelite analysis identified 14 quantitative trait loci relating to osteochondrosis. SNP identified additional quantitative trait loci in thoroughbreds, standardbreds and Hanoverians. Next-generation sequencing provided insight to genetic determination of equine OC.
Russell et al. (2017)	[42]	Osteochondrosis	Thoroughbred	Original Research	Retrospective cohort	Radiographs from thoroughbred yearlings between 2005 and 2013 (n = 1962), pedigrees from Australian Stud Book	Overall heritability of osteochondrosis ranged from 0 to 0.21. Heritability varied by affected joint. Environment of the dam affected some categories of osteochondrosis.
Jonsson et al. (2011)	[43]	Osteochondrosis	Swedish Warmblood	Original Research	Retrospective cohort	Equine hospital records (n = 879), and horses with recorded orthopedic problems (n = 3639) Heritability was analyzed using horses with pedigree information available (n = 3199)	Highlights utility of obtaining data from hospitals to perform heritability analyses on various inheritable disorders. Standardized documentation of genetic disorders assisted in future breeding decisions.
Grevenhof et al. (2009)	[44]	Osteochondrosis	Dutch Warmblood	Original Research	Retrospective cohort	Randomly selected yearlings from Royal Warmblood Studbook of the Netherlands (n = 881) descending from specific stallions (32)	At the animal level, overall osteochondrosis heritability was 0.23. Tarscocrural joints presented with highest heritability of osteochondrosis, and femoropatellar joints with the lowest. Selection against osteochondrosis was best performed when accounting status of all four joints (femoropatellar, tarscocrural, metacarpophalangeal and metatarsophalangeal joints) and differentiating between fragments and flattened bone contours.
Hilla et al. (2014)	[45]	Osteochondrosis	Hanoverian Warmblood	Original Research	Retrospective cohort	Standardized radiographic examination (n = 7396)	Heritability estimates for osteochondrosis at different joints were 0.17–0.34. Lower heritability estimates with osteochondral fragments, specifically in the fetlock joint. Heritability estimates indicated development of OC versus OCD in the hock, stifle, and fetlock could be treated as different traits.
Tessedre et al. (2012)	[46]	Osteochondrosis	French Trotter	Original Research	Genome Wide Association Study—Quantitative Trait Loci	French trotter progeny (n = 525) and sires with at least two progeny (98)	Multiple regions of the *Equus caballus* chromosomes were associated with overall global score for osteochondrosis. Four quantitative trait loci for global score were associated to a quantitative trait loci for hock and fetlock osteochondrosis, but not both. No quantitative trait loci overlapped between hock and fetlock osteochondrosis.
Wittwer et al. (2006)	[47]	Osteochondrosis	South German Coldblood (SGC)	Original Research	Radiographic examination	167 randomly sampled SGC, all registered in SCG Studbook and descendants of 30 SGC stallions	Sex of horse held significant influence over development of OC in the fetlock and hocks. Age of horse held significant influence over prevalence of OC in the hocks, as well as osteochondrotic findings in the distal tibia and palmar/plantar osseous fragments in the fetlocks. Radiographic signs of fetlock and hock OC increased significantly at one year of age.
Naccache et al. (2018)	[48]	Osteochondrosis	Multi	Review	Whole-genome scan	Previously published studies	Quantitative trait loci on multiple *Equus Caballus* genes (ECA) had correspondence across different breeds. In warmbloods, linkage disequilibrium between a locus on ECA 3 ands size gene LCORL were important in ODC development. Next-generation sequencing of DNA and RNA demonstrated powerful analysis to determine structural and genetic variants involved in the development of OC.
Lykkjen et al. (2014)	[49]	Osteochondrosis	Standardbred Trotters	Original Research	Retrospective cohort	Categorial data from radiographic studies performed in 1998 (1217) and 2007/2008, combined with sire threshold models n = 30)	Moderate to high heritability in tarscocrural OC and palmar/plantar first phalynx osteochondral fragments. Genetic correlations between OC and POF varied but were not significantly different from zero genetic correlation. No observation of effect from lineage or heterozygosity.
Dierks et al. (2007)	[50]	Osteochondrosis and osteochondrosis dissecans	Hanoverian Warmblood	Original Research	Genome Wide Association Scan	Hanoverian Warmbloods (n = 211) consisting of paternal half sibling families (n = 14)	Genome-wide significant quantitative trait loci were found on four equine chromosomes. Fetlock and hock OC demonstrated partial quantitative trait loci overlap on the same four chromosomes, indicating genetic linkage. On eight different chromosomes, quantitative trait loci reached chromosome-wide significance level.
Lampe et al. (2009)	[51]	Osteochondrosis and osteochondrosis dissecans	Hanoverian Warmblood	Original Research	Microsatellites (ECA18)	Hanoverian Warmbloods (n = 211) consisting of paternal half sibling families (n = 14)	Quantitative trait loci for osteochondrosis in fetlocks and/or hocks, or for OCD in hock joints between 74.9 and 82.5 Mb. Parathyroid hormone receptor 2 (PTH2R) demonstrated potential as a positional candidate gene within the quantitative trait loci for equine osteochondrosis. Identification of PTH2R as candidate gene for osteochondrosis due to regulating calcium metabolism.
Lampe et al. (2009)	[52]	Osteochondrosis and osteochondrosis dissecans	Hanoverian Warmblood	Original Research	Microsatellite (QTL on ECA16)	Hanoverian Warmbloods (n = 211) consisting of paternal half sibling families (n = 14)	Six microsatellites and one single-nucleotide polymorphism on QTL region between 24.26 and 42.41 Mb with significant association with hock OCD. Fetlock OC presented with similar findings between QTL 6.55 and 24.26 Mb on ECA16. No overlap between hock and fetlock QTL indicating different gene influence.
Dierks et al. (2010)	[53]	Osteochondrosis and osteochondrosis dissecans	Hanoverian Warmblood	Original Research	Microsatellite (QTL on ECA2)	Hanoverian Warmbloods (n = 211) consisting of paternal half-sibling families (n = 14)	Genes NCDN, FCN3 and MECR demonstrated associations between OC traits and SNPs on these genes. Significant effects for OC and OCD traits were found on NCDN-associated SNP. Identification of NCDN as a positional and functional candidate gene for OC.
Lampe et al. (2009)	[54]	Osteochondrosis and osteochondrosis dissecans	Hanoverian Warmblood	Original Research	Microsatellite (QTL on ECA5)	Hanoverian Warmbloods (n = 211) consisting of paternal half sibling families (n = 14)	Identification of collagen-type XXIV alpha as potential functional candidate gene. Overlap between quantitative trait loci of fetlock OC and OCD indicated same gene involvement. Identification of COL24A1 as a functional candidate for OC stemming from its involvement with disturbance of ossification.
Dierk et al. (2010)	[55]	Osteochondrosis and osteochondrosis dissecans	Hanoverian Warmblood	Original Research	Microsatellite (QTL on ECA4)	Hanoverian Warmbloods (n = 211) consisting of paternal half sibling families (n = 14)	Quantitative trait loci for hock and fetlock OC narrowed to 4.92 and 39.76 Mb on ECA4. Identification of quantitative trait loci specific to hock (3.63 to 6.24) and fetlock (7.42 to 13.10 and 56.15 to 39.76) osteochondrosis on ECA4.
Wittwer et al. (2007)	[56]	Osteochondrosis	South German Coldblood (SGC)	Original Research	Whole-genome scan	219 south German Coldbloods	10 chromosomes were found to have quantitative trait loci that had chromosome wide significance. 7 quantitative trait loci were identified specific to fetlock OC, and 1 quantitative trait locus was identified for both fetlock and hock OC on ECA18.
Orr et al. (2012)	[57]	Osteochondrosis	Dutch Warmblood	Original Research	Genome Wide Association Study	Pedigrees, radiographic data and blood samples from yearlings registered with the Royal Warmblood Studbook of the Netherlands (n = 811)	Associations between locations of chromosomes 3 and 10 indicating genomic regions linked to OC. Evidence of susceptibility on chromosome 10 provides evidence supporting standardbred trotter study identifying chromosome 10.
Lykkjen et al. (2010)	[58]	Osteochondrosis and osteochondrosis dissecans	Norwegian Standardbred Trotters	Original Research	Radiographic analysis and Genome Wide Association Analysis	Radiographic exams (n = 464) used to determine genome study candidates (n = 162, whole blood samples)	Identification of multiple SNPs with evidence of association with OCD in the tibial tarsal joint of Norwegian Standardbred Trotters. ECA 10 presented with two SNPs with the highest significance hits. Additional significant hits were found in quantitative trait loci on ECA 5, 27 and 28.
McCoy et al. (2016)	[59]	Osteochondrosis	American Standardbreed Trotters	Original Research	Genome Wide Association Study	American Standardbred yearlings from a single farm (n = 182) wholeblood or hair samples	Identification of risk loci on ECA14 for tarsal osteochondrosis. Risk loci were identified on ECA10 and 21.
Kemper et al. (2019)	[60]	Osteochondrosis	Multiple	Original Research	RNA squencing	Articular cartilage and subchondral bone from varying ages (Adult, n = 6; foal, n = 6)	1115 genes in articular cartilage were differentially expressed between age groups. 3574 genes in subchondral bone were differentially expressed between age groups. Domination of enriched pathways was found, representing cell cycle and signal transduction, and extracellular matrix organization and turnover.
Riddick et al. (2012)	[61]	Osteochondrosis	Multiple	Original Research	RNA sequencing, spatial proteomics and laser capture microdissection	Osteochondral and full-thickness cartilage samples bilaterally from distal femurs of foals aged 1–6 months (n = 15)	Alterations of cartilage maturation and ossification pathways led to the development of osteochondrosis. Downstream catabolic alterations are products of increased MMP3 and 13 expressions in osteochondrosis affected cartilage. Increased PDGF-A and MMP 13 gene expression was found in chondrocytes surrounding cartilage canals.
Mirams et al. (2009)	[62]	Osteochondrosis	Mixed	Original research	RT-PCR	Articular cartilage (foals, n = 14)	Increased expression of RUNX2, MMP13, type I collagen, and type X collagen mRNA in cartilage with lesions compared to healthy cartilage. Increased cells were present in clusters in cartilage with lesions versus healthy cartilage. No differences were noted in expression of type II collagen, connective tissue growth factor, aggrecan, Sox9, fibroblast growth factor and vascular endothelial growth in cartilage with lesions versus healthy cartilage.
Austbo et al. (2010)	[63]	Osteochondrosis	Standardbred, riding horse, thoroughbred	Original research	RAP-PCR	Articular cartilage (osteochondrosis predisposed foals (n = 9), control foals (n = 9)	Two genes, EST and TLK2, demonstrated significant upregulation when comparing compromised tissue with control tissue. Early TLK2 upregulation could reflect the difference in cell division from recovering tissue or increased growth rate from osteochondrosis. PAR-PCR in combination with RT-PCR provided a reliable screening method for identification of differentially expressed genes.
Verwilghen et al. (2009)	[64]	Osteochondrosis	Warmblood, other	Original research	Insulin Growth Factor 1 (IGF-I) assay	Radiographic and whole blood samples (n = 168)	No significant difference in IGF-I levels between warmbloods and other horse breeds. Significantly increased levels of IGF-I were found in affected group versus control. Significant positive correlation was found between horse weight and circulating IGF-I levels.
Valberg et al. (2003)	[65]	Skeletal Muscle Diease	N/A	Book	N/A	N/A	Multiple diagnostic methods of skeletal muscle disease were described, such as ancillary diagnostic test, exercise response tests, thermography, nuclear scintigraphy and ultrasound. Muscle disease was classified into multiple categories such as non-exercise associated rhabdomyolysis, exertional rhabdomyolysis, exertional myopathy with normal creatine kinase, muscle atrophy and muscle fasciculations. Each category had multiple methods of diagnosis and treatment.
McIlwraith et al. (2012)	[66]	Osteoarthritis	N/A	Review	N/A	N/A	The horse serves as an excellent model for development of osteoarthritis and treatment efficacy in humans.
Goodrich and Nixon (2006)	[67]	Osteoarthritis	N/A	Review	N/A	N/A	Methods to treat osteoarthritis include therapies aiming to inhibit further cartilage degeneration.
Thampi et al. (2022)	[68]	Osteoarthritis		Review	N/A	N/A	Viral or non-viral vectors can be used to deliver therapeutic molecules intra-articularly. Equine models have translational value to test therapeutics to treat osteoarthritis across species, including humans.
USDA (2001)	[69]	Lameness	N/A	Informational	N/A	N/A	Costs attributed to lameness in the equine industry were reported to be between $678 million and $1 billion annually (at the time of survey). Largest cost is attributed to loss of use of horses, followed by veterinary costs. Increased incidence of lameness combined with loss of use and veterinary costs resulted in lameness as the costliest condition compared with colic or other equine diseases such as equine protozoal myoencephalitis.
Schueter and Orth (2004)	[70]	Osteoarthritis	Multiple	Review	N/A	N/A	Molecular methods of understanding osteoarthritis and monitoring development were concluded to be improving. Non-invasive measures could be used to monitor effects of training on joint health.
Caron (2003)	[71]	Osteoarthritis	N/A	Book	N/A	N/A	Tissue making up synovial joints was summarized as synovium, synovial fluid, peri-articular soft tissues, articular cartilage and subchondral bone. There were multiple ways of monitoring development of osteoarthritis through synovial fluid changes, radiographic changes or more advanced imaging methods such as MRI or nuclear scintigraphy.
Keegan et al. (2007)	[72]	Lameness	N/A	Review	N/A	N/A	Subjective analyses were variable and there was a high degree of variability between observers. Kinetic and kinematic gait analysis provided clinicians with a consistent objective evaluation of limb lameness.
Kamm et al. (2013)	[73]	Osteoarthritis	Mixed breed	Original research	ELISA, PCR, and micorarray	Whole blood and synovial fluid (n = 24)	GRP, ADAMDEC1, RRM2, HCST, hUNC-93a were significantly different in WBCs of horses with osteoarthritis compared to non-osteoarthritis baseline levels. Gene expression profiles in cartilage, synovium, cartilage turnover proteins and WBCs demonstrated correlation. ADAMDEC1, RRM2 and hUNC-93A gene expression in WBCs showed correlation when measured through microarray and PCR assays.
Peffers et al. (2013)	[74]	Osteoarthritis	Mixed breed	Original research	RNA-seq and RT-PCR	Normal cartilage of metacarpophalangeal joint (n = 8)	Significant differences between 396 transcribed elements were seen from old and young cartilage. 303 genes were decreased, and 93 genes were increased levels in older cartilage. Genes with reduced expression relating to matrix synthesis enzymes, cytokines and growth factors and extracellular matrix were overrepresented in cartilage from old donors compared with young donors.
Welsh et al. (2013)	[75]	Musculoskeletal	Thoroughbred	Original research	Heritability analysis	Health records from Hong Kong Jockey Club (n = 5062)	Genetic correlation was found between fracture occurrence and osteoarthritis and suspensory ligament injury. Small to moderate heritability was estimated (0.01–0.2) for fracture, osteoarthritis, and suspensory ligament and tendon injury. Significant genetic components were described in development of fracture, osteoarthritis, tendon and ligament injuries.
Oki et al. (2008)	[76]	Musculoskeletal	Thoroughbred	Original research	Heritability analysis	Health records from Japan Racing Information System (n = 8198)	Significant effect of sex and age on development of superficial digital flexor tendon (SDFT) injuries with a higher occurrence in male horses versus female. Moderate heritability (0.17–0.19) indicating genetic contribution to risk. Selective breeding can help reduce soft tissue injury risk.
Goodship (1993)	[77]	Musculoskeletal	N/A	Review	N/A	N/A	Treatments (at the time of article publication) were limited, and none were found to have an effective cure for tendon injuries. The level of stretch occurring in the superficial digital flexor tendon of the thoroughbred horse was nearly identical to the amount of stretch that causes injury. Post-mortem tendon samples with known treatment history could further the development of novel treatment methods.
Tully et al. (2013)	[78]	Musculoskeletal	Thoroughbred	Original research	In silico gene assembly	Hair or whole blood samples (n = 2799), DNA extraction (n = 6), SNaPshot genotype (n = 38)	Decreased incidence of superficial digital flexor tendinopathy was found in heterozygous TNC BIEC2-696469 polymorphism horses compared to homozygous wild-type horses. Homozygous COL5A1 COL5A1_01 racehorses had a three-fold likelihood of developing SDF tendinopathy than those homozygous for wild-type alleles.

## Data Availability

Because no new data were created or analyzed in this study, data sharing is not applicable to this article.

## Abbreviations

The following abbreviations are used in this manuscript:

OA	Osteoarthritis
AQHA	American Quarter Horse Association
MSTN	Myostatin
OC	Osteochondrosis
WGS	Whole-Genome Sequencing
GWAS	Genome Wide Association Study
SDF	Superficial Digital Flexor
SNP	Single-Nucleotide Polymorphism
ECA	Equus Callabus Autosome
SNV	Single-Nucleotide Variant
IFG1	Insulin-like Growth Factor-1
MMP13	Matrix Metalloproteinase-13
MMP3	Matrix Metalloproteinase-3
RUNX2	Runt-Related Transcription Factor 2
PTH-RP	Parathyroid Hormone-Related Protein
